# Effect of Monthly, High‐Dose, Long‐Term Vitamin D Supplementation on Central Blood Pressure Parameters: A Randomized Controlled Trial Substudy

**DOI:** 10.1161/JAHA.117.006802

**Published:** 2017-10-24

**Authors:** John D. Sluyter, Carlos A. Camargo, Alistair W. Stewart, Debbie Waayer, Carlene M. M. Lawes, Les Toop, Kay‐Tee Khaw, Simon A. McG. Thom, Bernhard Hametner, Siegfried Wassertheurer, Kim H. Parker, Alun D. Hughes, Robert Scragg

**Affiliations:** ^1^ School of Population Health University of Auckland New Zealand; ^2^ Department of Emergency Medicine Massachusetts General Hospital Harvard Medical School Boston MA; ^3^ Department of General Practice The University of Otago Christchurch New Zealand; ^4^ Department of Public Health University of Cambridge United Kingdom; ^5^ International Centre for Circulatory Health Imperial College London London United Kingdom; ^6^ Center for Health & Bioresources AIT Austrian Institute of Technology Vienna Austria; ^7^ Department of Bioengineering Imperial College London London United Kingdom; ^8^ Institute of Cardiovascular Sciences University College London London United Kingdom

**Keywords:** arterial stiffness, blood pressure, randomized controlled trial, vitamin D, wave reflection, Clinical Studies, Hypertension, High Blood Pressure, Pharmacology, Treatment

## Abstract

**Background:**

The effects of monthly, high‐dose, long‐term (≥1‐year) vitamin D supplementation on central blood pressure (BP) parameters are unknown.

**Methods and Results:**

A total of 517 adults (58% male, aged 50–84 years) were recruited into a double‐blinded, placebo‐controlled trial substudy and randomized to receive, for 1.1 years (median; range: 0.9–1.5 years), either (1) vitamin D_3_ 200 000 IU (initial dose) followed 1 month later by monthly 100 000‐IU doses (n=256) or (2) placebo monthly (n=261). At baseline (n=517) and follow‐up (n=380), suprasystolic oscillometry was undertaken, yielding aortic BP waveforms and hemodynamic parameters. Mean deseasonalized 25‐hydroxyvitamin D increased from 66 nmol/L (SD: 24) at baseline to 122 nmol/L (SD: 42) at follow‐up in the vitamin D group, with no change in the placebo group. Despite small, nonsignificant changes in hemodynamic parameters in the total sample (primary outcome), we observed consistently favorable changes among the 150 participants with vitamin D deficiency (<50 nmol/L) at baseline. In this subgroup, mean changes in the vitamin D group (n=71) versus placebo group (n=79) were −5.3 mm Hg (95% confidence interval [CI], −11.8 to 1.3) for brachial systolic BP (*P*=0.11), −2.8 mm Hg (95% CI, −6.2 to 0.7) for brachial diastolic BP (*P*=0.12), −7.5 mm Hg (95% CI, −14.4 to −0.6) for aortic systolic BP (*P*=0.03), −5.7 mm Hg (95% CI, −10.8 to −0.6) for augmentation index (*P*=0.03), −0.3 m/s (95% CI, −0.6 to −0.1) for pulse wave velocity (*P*=0.02), −8.6 mm Hg (95% CI, −15.4 to −1.9) for peak reservoir pressure (*P*=0.01), and −3.6 mm Hg (95% CI, −6.3 to −0.8) for backward pressure amplitude (*P*=0.01).

**Conclusions:**

Monthly, high‐dose, 1‐year vitamin D supplementation lowered central BP parameters among adults with vitamin D deficiency but not in the total sample.

**Clinical Trial Registration:**

URL: http://www.anzctr.org.au. Unique identifier: ACTRN12611000402943.

**Clinical Perspective**

**What Is New?**

- Our randomized controlled trial of vitamin D supplementation used a unique combination of monthly, high‐dose vitamin D dosing for 1 year in a population‐based sample and assessed novel central blood pressure (BP) parameters not measured in previous trials.
- To our knowledge, this study is the first to show that monthly vitamin D supplementation lowers central BP parameters in vitamin D–deficient people.
- Our novel use of factor analysis showed that these reductions are attributed to 2 unique effects of vitamin D: decreases in maximum BP and pulse rate–related augmentation of the pressure waveform.

**What Are the Clinical Implications?**

- Because BP‐lowering effects were limited to vitamin D–deficient people, future trials should focus on this group.
- Because vitamin D lowered central BP parameters but had nonsignificant effects on brachial BP, future vitamin D assessments should include measuring central BP parameters to capture efficacy rather than rely solely on brachial BP.
- Although the lowering of central BP parameters in vitamin D–deficient people was sizeable and presumably is beneficial, randomized controlled trials are needed to confirm whether these effects translate into improvements in cardiovascular morbidity and mortality.

## Introduction

Systematic reviews of cohort studies have found that serum 25‐hyroxyvitamin D (25(OH)D) concentration correlates inversely with hypertension and cardiovascular events.^1^, ^2^ Because these associations were observational, it is not clear whether they are attributed to effects of vitamin D or to confounding factors such as physical activity and obesity. To investigate the causality and reversibility of low vitamin D and cardiovascular‐related end points, randomized controlled trials (RCTs) are required.

Several RCTs have examined the influence of vitamin D supplementation on brachial blood pressure (BP) and generally do not indicate a beneficial lowering effect.^3^, ^4^ In comparison, fewer RCTs have evaluated effects on variables derived from central BP waveforms.^5^ This is important because such measures, which include aortic systolic BP (SBP), augmentation index (AIx), pulse wave velocity (PWV), backward pressure amplitude, and excess pressure integral, predict cardiovascular events independently of or more strongly than brachial BP.^6^, ^7^, ^8^, ^9^ Moreover, RCTs show that antihypertensive medications have differential effects on these parameters despite similar influences on brachial BP,^10^, ^11^, ^12^ indicating that the latter has limited usefulness in capturing changes in central BP parameters. Because these findings suggest that measurements from central BP waveforms are different than those from brachial BP, it might be difficult to extrapolate results of vitamin D trials based on brachial BP to effects on central BP parameters.

Most prior RCTs of vitamin D supplementation on central BP parameters have reported no beneficial impact.^5^ These studies, however, were mostly short (≤6 months) and, consequently, might not have been long enough to find significant effects. Only a few RCTs have been long term (≥1 year),^13^, ^14^, ^15^, ^16^ but the daily dose equivalent (dose divided by days between each dose) of vitamin D used in these studies was submaximal (<1650 IU/day) and thus could both be suboptimal and underestimate possible effects at higher dosing regimens (eg, daily dose equivalent >3000 IU/day). Furthermore, the central BP parameters included in these studies were limited to SBP, diastolic BP (DBP), AIx, or PWV^13^, ^17^, ^18^, ^19^, ^20^, ^21^; they did not include other measures such as those derived from wave separation or reservoir wave analyses, which capture different aspects of arterial function.^8^, ^9^ Consequently, in an RCT, we examined the effect of long‐term, high‐dose vitamin D supplementation on a wide range of central BP parameters (and brachial BP for comparison).

## Methods

### Participants

This study was a prespecified analysis of a subsample of participants in the ViDA (Vitamin D Assessment) study who underwent safety‐related measurements for ≈1 year. The ViDA study was a randomized, double‐blinded, placebo‐controlled trial of the effect of vitamin D supplementation on health outcomes, with cardiovascular disease as the primary end point. Inclusion criteria were men and women aged 50 to 84 years and resident in Auckland, New Zealand. Exclusion criteria were (1) diagnosis of a terminal illness and/or in hospice care; (2) intending to leave New Zealand during the follow‐up period; (3) taking vitamin D supplements (including cod liver oil) of >600 IU daily if aged 50 to 70 years or >800 IU daily if aged 71 to 84 years; (4) history of renal stones, hypercalcemia, or medical conditions that can cause hypercalcemia; and (5) baseline serum calcium >2.50 mmol/L. Screening and baseline measurements took place between 2011 and 2012, with 5110 being randomized by computer to receive either vitamin D or placebo. Random assignment to one of the 2 treatment groups was made with random block sizes of 8, 10, or 12, within ethnic and 5‐year age groups. The randomization process was supervised by the study biostatistician (A.W.S.) to ensure that staff who collected the data were blinded to allocation. Ethics approval was provided by the New Zealand Multiregion Ethics Committee (MEC/09/08/082). Written, informed consent was obtained from each participant. This study was registered with the Australian New Zealand Clinical Trials Registry (ACTRN12611000402943). Full details of the study design, including a flowchart showing the number of people screened and excluded in the main ViDA study before the randomization of all 5110 participants, have been published elsewhere.^22^

### Vitamin D Intervention

Vitamin D_3_ (2.5 mg [100 000 IU]) or placebo softgel oral capsules, sourced from Tishcon Corp, were mailed to participants' homes. Two capsules were sent in the first mailing after randomization (ie, a 200 000‐IU bolus or placebo at the start of the intervention period), followed by a 2.5‐mg (100 000‐IU) capsule of vitamin D_3_ (or placebo) monthly throughout the remainder of the follow‐up period.

### Non–Arterial Function Measures

All measurements were carried out by trained staff using a standardized protocol. Questionnaires administered by interviewers were used to collect data on age, sex, ethnicity (defined by self‐identification), smoking, alcohol consumption, sun exposure, physical activity, diabetes mellitus, and use of vitamin D supplements and antihypertensive medications. The national medicine dispensing database was used to determine details of antihypertensive medications received at baseline and follow‐up, even for those who did not return for measurements. Without shoes and in light clothing, height was measured with a stadiometer to the nearest 0.1 cm, and weight was measured with digital scales to the nearest 0.1 kg. Body mass index was calculated as body weight (kg) divided by height (m)^2^.

Blood samples were collected at baseline and at 6 and 12 months of follow‐up, and plasma aliquots were stored frozen at −80°C. Serum 25(OH)D (combining D_2_ and D_3_) concentration was measured in these aliquots (baseline and follow‐up samples were measured in the same batch for each participant) by liquid chromatography–tandem mass spectrometry (ABSciex API 4000) at a laboratory participating in the DEQAS (Vitamin D External Quality Assessment Scheme) program (http://www.deqas.org).

### Arterial Function Measurements

Arterial function measurements were made at baseline and at 1‐year follow‐up. After 15 minutes of rest and while sitting, brachial BP (±1 mm Hg) was measured 3 times with an Omron T9P oscillometric device (Omron Healthcare) above the cubital fossa of the left arm. The mean of the 2 closest measurements was used for analyses. Hypertensive participants were defined as those who had brachial SBP ≥140 mm Hg or brachial DBP ≥90 mm Hg and/or who were receiving antihypertensive medications (determined from the questionnaires and the national medicine dispensing database).

Suprasystolic oscillometry was carried out using a BP+ device (Uscom [formerly known as the R6.5 cardiovascular monitor; Pulsecor]), with an appropriately sized cuff positioned over the left upper arm. The BP+ device has been shown (1) to yield central SBPs that are highly correlated with those assessed by catheter measurement at the ascending aorta or aortic arch^23^ and (2) to measure central SBP with good intratest and intertest reliability.^24^ To improve the quality of the waveforms used in analyses, we decided a priori to exclude readings with a signal‐to‐noise ratio <3 dB (below acceptable).

In addition to aortic SBP and DBP, several parameters that predict cardiovascular events independently of brachial BP were calculated from the aortic pressure waveform.^6^, ^8^, ^9^, ^25^, ^26^, ^27^, ^28^, ^29^ AIx (%),^6^ an index of arterial stiffness and wave reflection,^30^ was calculated from the aortic pressure waveform using custom‐written Matlab software (Mathworks). Aortic PWV^7^ was calculated from validated algorithms.^25^, ^26^ Aortic pressure was separated into reservoir and excess components using custom‐written Matlab software. Reservoir pressure was calculated from pressure measurements only, as described elsewhere.^8^ Peak reservoir pressure was calculated as the maximum of the reservoir pressure waveform.^27^ Excess pressure was calculated as measured pressure minus reservoir pressure.^8^ The integral of the excess and reservoir pressure waveforms (area under these) over the cardiac cycle was used to calculate excess pressure integral and reservoir pressure integral, respectively. The former measures pressure associated with excess ventricular work.^8^ Aortic pressure was separated into forward‐ and backward‐traveling pressure waves using wave separation analysis.^27^ Their amplitudes—forward pressure amplitude and backward pressure amplitude^9^, ^29^—were then calculated by a technique that yields values similar to those obtained using true aortic flow waves measured by Doppler ultrasound.^31^ Wave intensity analysis was used to calculate wave reflection index.^28^

### Statistical Analyses

Data were analyzed using SAS version 9.3 (SAS Institute). Baseline group differences in characteristics and differential missingness of data were assessed with ANOVA (for continuous variables) and the chi‐square test (for categorical variables). We examined changes from baseline to follow‐up in (1) the number of antihypertensive drugs, with the paired *t* test and Wilcoxon signed‐rank test, and (2) the proportion receiving antihypertensive medication, with the McNemar test. Treatment group differences at follow‐up were assessed with Monte Carlo estimates of the Fisher exact test (for the number of antihypertensive drugs) and the chi‐square test (for the proportion on antihypertensive medication). Variables that were positively skewed (excess pressure integral and wave reflection index) were log‐transformed. Factor analysis with varimax rotation was applied to BP parameters to reduce these to fewer, uncorrelated factors that represent distinct attributes that explain a high fraction of the variability in the original variables. These factors were extracted by the method of principal components, and only principal components that accounted for more than the variance of 1 variable (eigenvalue >1) were retained and used in subsequent analyses. Factor loadings (correlations between factors and original variables) of ≥0.3 were considered significant.^32^ On an intention‐to‐treat basis, general linear mixed models were used to assess the effect of vitamin D supplementation on 25(OH)D and BP parameters (adjusted for age, sex, and ethnicity) with repeated time incorporated using an unstructured correlation structure, using PROC MIXED. This analysis method handles missing data by fitting a statistical model over all available observations without introducing bias. Specifically, to test whether the change from baseline differed across the treatment groups, we examined the interaction between treatment group and time.

Deseasonalized (season‐adjusted) baseline 25(OH)D levels were calculated for each participant from the midpoint between the estimated maximum and minimum 25(OH)D levels over a calendar year from their individual measured baseline 25(OH)D and date of blood collection, using a sinusoidal model with parameters derived from baseline values for all participants in the main ViDA study.^33^ Vitamin D deficiency was defined as having a deseasonalized 25(OH)D <50 nmol/L.^33^

In addition to performing analyses in the total sample, we decided a priori to perform subgroup analyses among vitamin D–deficient persons. This is because nonlinear relationships between 25(OH)D concentration and health outcomes, including mortality^34^, ^35^ and CVD,^2^ suggest that adverse effects associated with low vitamin D status are greatest in vitamin D–deficient persons, indicating that vitamin D supplementation could be more effective in such individuals. In further prespecified analyses, we examined 3‐way interactions among vitamin D deficiency (present or absent), treatment group, and time so as to test whether the effects of vitamin D were different in those with and without vitamin D deficiency and supplemented this with a subgroup analysis among people without vitamin D deficiency.

Because of the influence of antihypertensive medications on BP, we examined 3‐way interactions between antihypertensive treatment (presence or absence), treatment group (vitamin D or placebo), and time so as to test whether the effects of vitamin D were different in those who received and did not receive antihypertensive therapy. The ViDA study was originally powered to detect a clinically relevant reduction in cardiovascular events (primary outcome), as described elsewhere.^22^ For the current substudy, with 90% power and at the 5% significance level (2‐tailed), the detectable differences in standard deviations of BP parameters were 0.3 in the total sample and 0.5 in the vitamin D–deficient sample. Scatter plots fitted with smoothed curves^36^ (using PROC LOESS) revealed in the vitamin D group that change in central BP parameters appeared to vary with baseline deseasonalized 25(OH) up to ≈65 nmol/L in several cases and remained relatively constant beyond this threshold. Consequently, we used Pearson correlation coefficients to summarize these associations in people with baseline deseasonalized 25(OH)D <65 nmol/L. Correlations between changes in deseasonalized 25(OH)D and changes in BP parameters were also summarized with Pearson correlation coefficients. Robust estimates (95% confidence intervals [CIs]) of these correlation coefficients were calculated using 1000 bootstrap samples. Statistical significance was set at *P*<0.05 (2‐sided). No *P* value correction was applied to account for multiple hypothesis tests, as we did not want to miss any important findings.^37^

## Results

**Figure 1:**
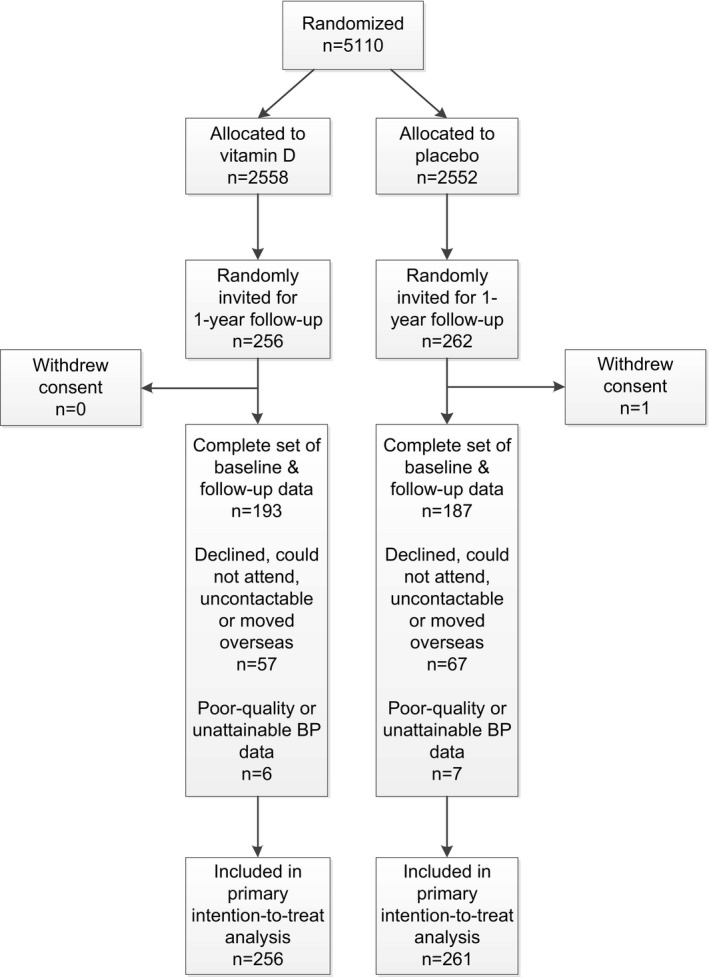
Flowchart showing the number of randomized participants who were excluded and those with complete sets of both baseline and 1‐year follow‐up measurements. BP indicates blood pressure.

Figure 1 shows the study flow diagram. Of the 5110 participants randomized in the main ViDA study, 518 (10%) were randomly selected and invited to partake in the current substudy. Of these, 1 withdrew consent (analysis of data disallowed) and was not included in any further analysis. Of the remaining 517, a complete set of both baseline and 1‐year follow‐up measurements was available for 380 and missing for 137 (26%): 124 did not attend the follow‐up interview (declined to attend, could not attend, could not be contacted, or moved overseas), and 13 had arterial BP waveform data that were poor quality (signal‐to‐noise ratio <3 dB) or unobtainable (unable to get a reading). Most (57%) of 137 missing follow‐up cases were due to people indicating at their baseline assessment that they did not wish to return for a follow‐up interview, ruling out the possibility of bias from loss to follow‐up (missingness due to changes in BP‐related health) in this group. All of the abovementioned 517 participants were included in the intention‐to‐treat analysis. Altogether, the percentage of the intention‐to‐treat sample that had missing follow‐up data did not differ across the 2 treatment groups (*P*=0.38). Furthermore, this missingness was unrelated to baseline BP parameters such as brachial SBP (*P*=0.87).

The regimens of antihypertensive medications (categorized by their classes) by treatment group at baseline and follow‐up are shown in Tables S1 (total sample) and S2 (vitamin D–deficient sample). In the total sample, neither the number of drugs nor the proportion receiving antihypertensive medication changed from baseline to follow‐up in either the vitamin D or the placebo group (*P* values varying from 0.31 to 0.68). This was also true in the vitamin D–deficient sample (*P* values of 0.09–0.80). Furthermore, in each sample, neither the number of drugs nor the proportion receiving antihypertensive medication differed across treatment groups at follow‐up (*P* values of 0.14–0.64).

Of the entire intention‐to‐treat sample (total sample), 256 received vitamin D and 261 received placebo. Among vitamin D–deficient people, 71 and 79 received vitamin D and placebo, respectively. Baseline characteristics of these participants by treatment group are given in Table 1. In the total sample, the mean age was 65.0 years (range: 50–84 years), 56% were male, and 75% were of European or other ethnicity (96% of whom had European ancestry). Just under half had smoked cigarettes or used tobacco (with most being former smokers), 14% reported <1 h/day of sun exposure, 11% took vitamin D supplements (within the study eligibility criteria) and nearly two thirds were hypertensive. The mean concentrations of observed and deseasonalized 25(OH)D were 63 nmol/L (SD: 25) and 66 nmol/L (SD: 24), respectively, with 29% of people having a deseasonalized 25(OH)D of <50 nmol/L (indicative of vitamin D deficiency). In comparison, the vitamin D–deficient sample—with mean observed and deseasonalized 25(OH)D concentrations of 36 nmol/L (SD: 10) and 39 nmol/L (SD: 8), respectively—had a greater percentage of non‐Europeans (49%). The time period between randomization and follow‐up averaged 1.1 years (both mean and median) and ranged from 0.9 to 1.5 years.

**Table 1:** Baseline Comparison of Participants Across Treatment Groups

Variable	Total Sample		Vitamin D‐Deficient Sample^a^	
	Vitamin D (n=256)	Placebo (n=261)	Vitamin D (n=71)	Placebo (n=79)
Age, y^b^	64.5±8.3	65.5±8.8	63.3±8.6	64.7±9.1
Male sex, %	60	52	56	48
Ethnicity				
European/other, %	73	78	49	52
Maori, %	7	7	14	8
Pacific, %	12	8	17	22
South Asian, %	8	7	20	19
Smoking				
Nonsmoker, %	53	50	56	58
Former smoker, %	37	42	34	38
Current smoker, %	9	8	10	4
Alcohol consumption				
Nondrinker, %	8	7	12	13
Former drinker, %	11	8	17	18
Current drinker, %	82	86	71	70
Sun exposure, h/d				
<1, %	14	13	20	19
1–2, %	53	52	48	62
>2, %	32	35	32	19
Vigorous physical activity, h/wk				
None, %	39	39	55	49
1–2, %	25	27	26	26
>2, %	37	35	20	26
Diabetes mellitus, %	2	3	3	6
Vitamin D supplements, %	11	11	4	6
Hypertension, %	66	63	65	77
Antihypertensive medication, %	44	39	48	56
Body mass index, kg/m^2^ b	28.8±5.2	28.6±5.2	30.8±6.9	29.7±6.2
25‐hydroxyvitamin D				
Observed^b^	62.1±24.7	63.1±24.6	35.2±10.2	36.5±9.9
Deseasonalized^b^	65.8±23.8	66.0±24.1	38.1±8.6	38.9±8.0
Deseasonalized <50 nmol/L, %	28	30	100	100
Time from randomization to follow‐up, d^b^	401±29	402±30	398±32	401±32

a Those with deseasonalized 25‐hydroxyvitamin D <50 nmol/L.

b Values are mean±SD.

**Figure 2:**
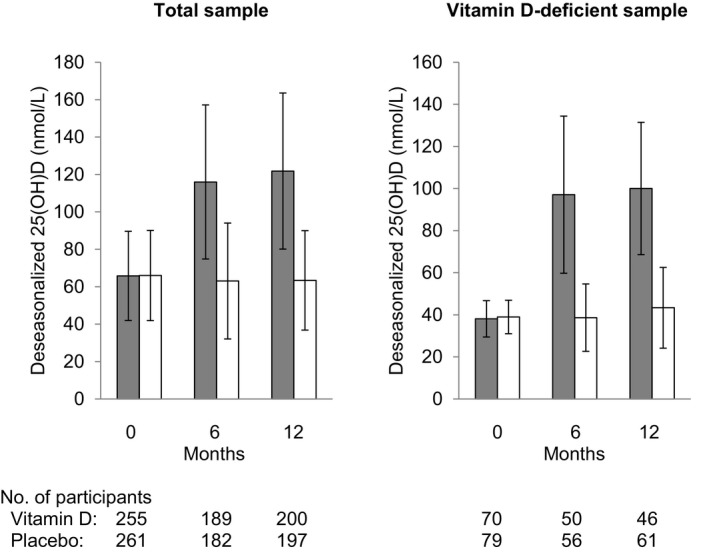
Bar graphs showing deseasonalized 25(OH)D concentration (nmol/L) at baseline and follow‐up (6 and 12 mo) in the vitamin D (gray bars) and placebo (white bars) groups. The bars represent mean±SD.

Figure 2 shows the deseasonalized 25(OH)D concentration at baseline and follow‐up visits by treatment group. The change from baseline in the vitamin D group with respect to placebo at 6‐ and 12‐month follow‐up, respectively, was 51 nmol/L (95% CI, 44–57) and 58 nmol/L (95% CI, 51–64), respectively, for the total sample and 56 nmol/L (95% CI, 45–66) and 58 nmol/L (95% CI, 48–68) nmol/L, for the vitamin D‐deficient sample (all *P*<0.001).

Table 2 shows BP waveform parameters at baseline and follow‐up by intervention group in the total sample (n=517). The effects of vitamin D compared with placebo on the parameters were in the positive direction for pulse rate, AIx, and log_e_(wave reflection index) and consistently in the negative direction for the remaining variables; however, observed changes were small and not statistically significant. In addition, these effects were not moderated by antihypertensive therapy (*P* values varying from 0.29 to 0.96).

**Table 2:** Arterial Function Measures at Baseline and Follow‐up (Adjusted for Age, Sex, and Ethnicity) by Treatment Group in the Total Sample (N=517)

Variable	Mean (SD)				Change From Baseline, Vitamin D Minus Placebo	
	Vitamin D Group (n=256)		Placebo Group (n=261)			
	Baseline	Follow‐up	Baseline	Follow‐up	Mean (95% CI)	*P* Value
Pulse rate, beats/min	63.0 (10.0)	65.7 (9.5)	63.9 (11.0)	65.7 (11.8)	0.9 (−0.7 to 2.6)	0.27
Brachial SBP, mm Hg	137.7 (18.4)	128.9 (16.1)	137.7 (16.8)	131.0 (18.9)	−2.1 (−5.2 to 0.9)	0.17
Brachial DBP, mm Hg	78.4 (10.6)	73.7 (9.9)	78.7 (9.7)	74.8 (9.9)	−0.8 (−2.5 to 0.8)	0.32
Aortic SBP, mm Hg	140.1 (18.4)	131.1 (16.2)	139.7 (17.8)	132.9 (20.2)	−2.2 (−5.4 to 0.9)	0.17
Aortic DBP, mm Hg	72.0 (6.6)	69.5 (6.1)	72.2 (6.0)	70.2 (6.1)	−0.5 (−1.5 to 0.6)	0.41
Augmentation index, %	30.0 (12.1)	27.0 (11.2)	29.9 (13.1)	26.8 (12.6)	0.0 (−2.4 to 2.5)	0.98
Pulse wave velocity, m/s	9.3 (1.7)	9.2 (1.6)	9.3 (1.7)	9.3 (1.9)	−0.1 (−0.2 to 0.0)	0.18
Peak reservoir pressure, mm Hg	124.3 (17.8)	116.5 (15.1)	124.2 (16.4)	118.4 (18.5)	−2.0 (−5.1 to 1.1)	0.21
Peak excess pressure, mm Hg	28.5 (8.3)	25.8 (7.9)	28.1 (8.5)	26.1 (8.3)	−0.7 (−2.5 to 1.0)	0.40
Reservoir pressure integral, mm Hg/s	92.1 (18.3)	83.0 (16.7)	91.7 (21.0)	85.4 (21.5)	−2.8 (−6.1 to 0.5)	0.10
Log_e_(excess pressure integral, mm Hg/s)	1.57 (0.38)	1.40 (0.42)	1.54 (0.43)	1.39 (0.43)	−0.02 (−0.11 to 0.06)	0.59
Backward pressure amplitude, mm Hg	28.6 (7.3)	25.3 (6.3)	28.5 (7.5)	25.9 (8.3)	−0.8 (−2.0 to 0.5)	0.25
Forward pressure amplitude, mm Hg	40.0 (8.4)	36.8 (7.9)	39.5 (8.5)	37.5 (9.6)	−1.2 (−2.7 to 0.4)	0.14
Log_e_(wave reflection index), ×10^−2^	−120.6 (35.0)	−124.5 (28.8)	−118.1 (35.9)	−125.7 (35.7)	3.7 (−4.3 to 11.8)	0.36

CI indicates confidence interval; DBP, diastolic blood pressure; SBP, systolic blood pressure.

These analyses were repeated in participants with vitamin D deficiency at baseline (n=150; Table 3). Pulse rate change did not significantly differ across the 2 treatment groups (*P*=0.05). With respect to placebo, vitamin D reduced aortic SBP (−7.5 mm Hg), AIx (−5.7%), PWV (−0.3 m/s), peak reservoir pressure (−8.6 mm Hg), reservoir pressure integral (−9.8 mm Hg/s), backward pressure amplitude (−3.6 mm Hg), and forward pressure amplitude (−3.3 mm Hg; all *P*<0.05). The effects (vitamin D minus placebo) on the remaining parameters, including brachial SBP and DBP, were consistently in the negative direction, although not statistically significant (*P*>0.05). Antihypertensive treatment did not moderate any of these effects (*P* values of 0.15–0.92). Factor analysis of changes (follow‐up minus baseline) in these parameters yielded 4 factors (Table S3), but only factors 1 and 2 differed across the 2 intervention groups (Table S4) and, as evident from their loadings (Table S3), predominantly accounted for the significant, between‐group changes in individual BP parameters (Table 3). In other words, compared with placebo, vitamin D reduced factors 1 (representing maximum BP) and 2 (representing slower pulse rate and raised augmentation of the pressure waveform; Table S4).

**Table 3:** Arterial Function Measures at Baseline and Follow‐up (Adjusted for Age, Sex, and Ethnicity) by Treatment Group Among Those With Baseline Vitamin D Deficiency (Deseasonalized 25(OH)D <50 nmol/L; n=150)

Variable	Mean (SD)				Change From Baseline, Vitamin D Minus Placebo	
	Vitamin D Group (n=71)		Placebo Group (n=79)			
	Baseline	Follow‐up	Baseline	Follow‐up	Mean (95% CI)	*P* Value
Pulse rate, beats/min	62.9 (10.9)	65.7 (9.9)	66.2 (13.3)	65.4 (12.8)	3.5 (−0.0 to 7.1)	0.05
Brachial SBP, mm Hg	137.4 (16.8)	125.5 (13.0)	139.4 (18.2)	132.8 (20.1)	−5.3 (−11.8 to 1.3)	0.11
Brachial DBP, mm Hg	78.9 (10.7)	72.8 (9.2)	80.0 (11.3)	76.6 (10.8)	−2.8 (−6.2 to 0.7)	0.12
Aortic SBP, mm Hg	139.8 (18.5)	127.1 (14.1)	141.1 (18.6)	136.0 (21.4)	−7.5 (−14.4 to −0.6)	0.03
Aortic DBP, mm Hg	72.2 (6.4)	68.9 (5.5)	73.2 (7.0)	71.3 (6.5)	−1.3 (−3.7 to 1.0)	0.25
Augmentation index, %	29.7 (13.6)	22.9 (8.7)	29.1 (13.7)	28.1 (14.5)	−5.7 (−10.8 to −0.6)	0.03
Pulse wave velocity, m/s	9.2 (1.8)	8.9 (1.5)	9.2 (1.9)	9.3 (2.0)	−0.3 (−0.6 to −0.1)	0.02
Peak reservoir pressure, mm Hg	125.2 (18.0)	112.3 (12.4)	125.2 (17.9)	120.9 (20.1)	−8.6 (−15.4 to −1.9)	0.01
Peak excess pressure, mm Hg	26.5 (6.8)	25.7 (8.4)	28.6 (8.7)	27.0 (7.9)	0.7 (−2.8 to 4.1)	0.70
Reservoir pressure integral, mm Hg/s	93.0 (19.1)	81.2 (16.2)	89.5 (21.2)	87.5 (23.1)	−9.8 (−16.2 to −3.3)	0.003
Log_e_(excess pressure integral, mm Hg/s)	1.51 (0.39)	1.38 (0.47)	1.54 (0.42)	1.45 (0.43)	−0.04 (−0.22 to 0.14)	0.65
Backward pressure amplitude, mm Hg	28.7 (8.0)	23.5 (6.0)	28.4 (7.7)	26.7 (9.1)	−3.6 (−6.3 to −0.8)	0.01
Forward pressure amplitude, mm Hg	39.7 (8.8)	34.7 (6.3)	40.1 (8.7)	38.4 (9.9)	−3.3 (−6.4 to −0.2)	0.04
Log_e_(wave reflection index), ×10^−2^	−117.7 (34.2)	−130.6 (31.3)	−122.6 (41.8)	−125.1 (50.2)	−10.3 (−29.2 to 8.6)	0.28

CI indicates confidence interval; DBP, diastolic blood pressure; SBP, systolic blood pressure.

Further analysis showed that vitamin D (with respect to placebo) caused greater reduction of AIx (*P*=0.009), PWV (*P*=0.0498), peak reservoir pressure (*P*=0.01), reservoir pressure integral (*P*=0.01), backward pressure amplitude (*P*=0.01), and loge(wave reflection index) (*P*=0.047) in participants with vitamin D deficiency than in those without. As a reflection of this, the effects of vitamin D (versus placebo) in those with vitamin D deficiency (Table 3) were larger than in those without (Table S5).

Correlations between deseasonalized 25(OH)D (baseline and change) and change in BP parameters in the vitamin D group are shown in Table S6. At <65 nmol/L, baseline deseasonalized 25(OH)D was positively correlated with changes in aortic SBP, AIx, PWV, peak reservoir pressure, and forward pressure amplitude (*r*=0.18–0.24), indicating larger reductions with decreasing 25(OH)D. Among those with vitamin D deficiency (deseasonalized 25(OH)D <50 nmol/L) at baseline, 25(OH)D change was negatively correlated (*r*=−0.23 to −0.31) with change in several parameters, which included all of those shown in Table 3 that were reduced by vitamin D supplementation.

## Discussion

This randomized, double‐blinded, placebo‐controlled trial demonstrated that monthly high‐dose (daily dose equivalent >3300‐IU/day) vitamin D supplementation over an average period of 1.1 years had little effect on central and brachial BP parameters in the total sample. However, among people with vitamin D deficiency at baseline, it resulted in presumably beneficial reductions in several central BP parameters that, as evident from the factor analysis results, are attributed to 2 unique effects of vitamin D: decreases in maximum BP and pulse rate–related augmentation of the pressure waveform.

The absence of antihypertensive effects in the total sample (73% without vitamin D deficiency) concurs with several previous vitamin D trials that similarly comprised mostly participants without vitamin D deficiency.^5^ We extended these past trial findings, as our study used a unique combination of monthly high‐dose vitamin D dosing for 1 year in a population‐based sample and assessed novel parameters not measured in previous trials (eg, excess pressure integral and backward pressure amplitude). For instance, only a few prior RCTs used monthly ≥100‐kIU dosing (as our study did) but for not more than 6 months.^17^, ^21^, ^38^

To our knowledge, this study is the first to show that monthly vitamin D supplementation reduces central BP variables in persons with vitamin D deficiency. Two RCTs of vitamin D–deficient participants found beneficial effects of vitamin D supplementation on AIx but not on PWV.^39^, ^40^ Compared with our study, these RCTs had shorter follow‐up periods (<6 months) and different dosing regimens (daily^39^ or a single intramuscular dose^40^) and did not measure other parameters (eg, peak reservoir pressure) that were modified beneficially by vitamin D in our study. In contrast, other RCTs in vitamin D–deficient participants found that vitamin D supplementation did not result in improvements in any central BP parameters: PWV, AIx, SBP, DBP, or pulse pressure.^14^, ^41^, ^42^, ^43^ Possible reasons for this discrepancy are that most of these studies were smaller (n≤62) and of shorter duration (<6 months),^41^, ^42^, ^43^ whereas the remaining study administered half the vitamin D dose (50 000 IU monthly) that we used.^14^ Because we observed beneficial effects in vitamin D–deficient participants but not in the total sample, and several parameters decreased more in participants with vitamin D deficiency than in those without, this implies that vitamin D trials in people with normal vitamin D levels may underestimate effects among those with vitamin D deficiency. Consequently, future trials should focus on vitamin D‐deficient persons.

A mechanism by which vitamin D may lower BP is through impact on the renin–angiotensin system,^44^ indicating that it could be blunted by antihypertensive medications; however, this may not be the only mechanism, as antihypertensive therapy did not moderate the vitamin D effects in our study. Antihypertensive effects could also occur through improvement in endothelial function and a reduction in vascular tone.^44^ We showed that this could be associated with a decrease in arterial stiffness, as PWV was lowered by vitamin D (Table 3). Furthermore, we propose that this reduction in vascular tone could improve impedance matching at arterial pressure wave reflection sites, thereby reducing the magnitude of reflected arterial pressure waves, as has been suggested to occur with antihypertensive vasodilator drugs.^11^, ^45^ This wave reflection effect is supported by vitamin D lowering backward pressure amplitude (Table 3).

In the vitamin D–deficient sample, the magnitude of each significant intervention effect as a percentage of the average BP parameter value for a person was sizeable. For example, the intervention effect for backward pressure amplitude (−3.6 mm Hg) as a percentage of the mean baseline value for this parameter in the vitamin D group (28.7 mm Hg; Table 3) was 13%. Importantly, these effects would translate into clinically meaningful reductions in cardiovascular risk.^6^, ^7^, ^8^, ^9^, ^27^, ^29^ Given that, for example, a 10% increase in central AIx is associated with a relative risk for cardiovascular outcomes of 1.318,^6^ a 5.7% decrease (net vitamin D effect; Table 3) would correspond to a theoretical relative risk reduction of up to 17%. These beneficial risk reductions would be partly cumulative because the factor analysis revealed that the BP parameter changes reflect 2 unique effects rather than a single one.

In the main ViDA study, vitamin D supplementation had no apparent effect on cardiovascular events.^46^ This does not necessarily mean that the reductions in central BP parameters observed in the current substudy (Table 3) do not translate into improvements in cardiovascular morbidity and mortality. It may be that any improvements (eg, ≤17% relative risk reduction; mentioned in the previous paragraph) were smaller than those that could be detected in the main ViDA study (21% reduction at up to 80% power^46^).

Although effects on brachial BP were in the negative (lowering) direction, they were non‐significant, unlike those for central BP parameters (Table 3). This suggests that vitamin D trials based on brachial BP^3^ underestimate effects on central BP parameters. Consequently, future trials should include measuring central BP parameters to capture efficacy rather than rely solely on brachial BP.

Our study was population‐based, which enhances the generalizability of our findings. As for the limitations, the missingness of the intention‐to‐treat sample (Figure 1) raises the possibility that selection bias could influence the study findings. As reported, however, this missingness did not differ across the treatment groups and was unrelated to BP parameters. A longer follow‐up period may have enabled us to gain better insight into the long‐term effects of the intervention. Although larger than prior RCTs of vitamin D and central BP parameters,^13^, ^17^, ^18^, ^19^, ^20^, ^21^ our statistical power was limited (especially in the vitamin D–deficient sample), which may explain the lack of statistical significance of some treatment effects. Finally, the use of multiple outcomes increases the likelihood that at least some of our significant findings could be due to chance. However, the treatment effects were in line with observational research^1^, ^2^, ^34^ and consistently unidirectional. In addition, if study conclusions are based on factor analysis results only (which summarize the individual relationships), fewer comparisons are involved.

Although our analyses were carried out in subsamples of an RCT (Figure 1), we do not expect there to be important imbalances in participant characteristics at baseline for several reasons. First, the selection of our total analysis sample from the main ViDA study was random. Second, the selection of subgroups from the total sample would not differ by treatment group because everyone was randomized the same way. Third, effects of any imbalances in age, sex, and ethnicity would have been minimized because these demographic variables were adjusted for in analyses. Fourth, baseline imbalances can be reduced by stratifying the study randomization by subgroup variables.^47^ Regarding that, stratifying randomization by 25(OH)D concentration per se was not carried out, but (1) some have proposed that this is not required for prespecified subgroup analyses (eg, our study),^48^ and (2) its effect on reducing imbalances in subgroups would have been partially captured because we stratified randomization by age and ethnicity, which predict 25(OH)D.^49^ Fifth, imbalances are more influential with small sample sizes (smaller than ours).^47^, ^48^ Finally, the above points are reflected in the important finding that characteristics at baseline were similar between the treatment groups (Table 1).

In summary, monthly high‐dose vitamin D supplementation for just slightly >1 year, which increased serum 25(OH)D concentration by >50 nmol/L with respect to placebo, had little effect on BP parameters in the total sample. In the vitamin D–deficient sample, however, this supplementation did not significantly change brachial BP but had clinically relevant, beneficial effects on central BP parameters. RCTs (of adequate statistical power) are needed to confirm whether these effects translate into improvements in cardiovascular morbidity and mortality.

## Sources of Funding

The Health Research Council of New Zealand (HRC) and Accident Compensation Corporation of New Zealand funded this study. HRC supported Sluyter with a postdoctoral fellowship.

## Disclosures

None.

## Supporting information


**Table S1.** Antihypertensive Medication Regimens in the Total Sample (N=517)^1^
**Table S2.** Antihypertensive Medication Regimens in the Vitamin D‐Deficient Sample (n=150)^1^
**Table S3.** Eigenvalues of the Correlation Matrix and Loadings in the Factor Analysis (Varimax Solution) for Change (Follow‐up Minus Baseline) in Arterial Waveform Parameters Among Those With Baseline Vitamin D Deficiency (Deseasonalized 25(OH) <50 nmol/L)
**Table S4.** Changes (Follow‐up Minus Baseline) in Factor Analysis Variables (Adjusted for Age, Sex, and Ethnicity) by Treatment Group Among Those With Baseline Vitamin D Deficiency (Deseasonalized 25(OH) <50 nmol/L)
**Table S5.** Arterial Function Measures at Baseline and Follow‐Up (Adjusted for Age, Sex, and Ethnicity) by Treatment Group Among Those Without Baseline Vitamin D Deficiency (Deseasonalized 25(OH) ≥50 nmol/L; n=367)
**Table S6.** Correlations of Deseasonalized 25(OH)D Concentration (Baseline and Change*) With Changes* in Arterial Function Measures in the Vitamin D Group
