# Supplementary material for: Effect of Monthly, High‐Dose, Long‐Term Vitamin D Supplementation on Central Blood Pressure Parameters: A Randomized Controlled Trial Substudy
Source: J Am Heart Assoc. 2017 Oct 24;6(10):e006802. doi: 10.1161/JAHA.117.006802 (PMC5721873; doi:10.1161/JAHA.117.006802)
Supplement: Supplementary file 1 — Table S1. Antihypertensive Medication Regimens in the Total Sample (N=517)1 Table S2. Antihypertensive Medication Regimens in the Vitamin D‐Deficient Sample (n=150)1 Table S3. Eigenvalues of the Correlation Matrix and Loadings in the Factor Analysis (Varimax Solution) for Change (Follow‐up Minus Baseline) in Arterial Waveform Parameters Among Those With Baseline Vitamin D Deficiency (Deseasonalized 25(OH) <50 nmol/L) Table S4. Changes (Follow‐up Minus Baseline) in Factor Analysis Variables (Adjusted for Age, Sex, and Ethnicity) by Treatment Group Among Those With Baseline Vitamin D Deficiency (Deseasonalized 25(OH) <50 nmol/L) Table S5. Arterial Function Measures at Baseline and Follow‐Up (Adjusted for Age, Sex, and Ethnicity) by Treatment Group Among Those Without Baseline Vitamin D Deficiency (Deseasonalized 25(OH) ≥50 nmol/L; n=367) Table S6. Correlations of Deseasonalized 25(OH)D Concentration (Baseline and Change*) With Changes* in Arterial Function Measures in the Vitamin D Group [file JAH3-6-e006802-s001.pdf]

Supplemental Material

Table S1. Antihypertensive medication regimens in the total sample; n=517[1]

Antihypertensive regimen	n			
	Vitamin D group		Placebo group	
	Baseline	Follow-up	Baseline	Follow-up
**0 drugs**	**160**	**154**	**171**	**167**
**1 drug**	**45**	**48**	**32**	**36**
ACEI	22	17	15	14
αB	1	4	0	1
ARB	4	5	5	4
βB	5	4	4	6
CCB	7	11	6	8
D	6	7	2	3
**2 drugs**	**31**	**36**	**36**	**32**
ACEI + αB	0	0	1	2
ACEI + ARB	0	1	0	0
ACEI + βB	4	5	7	6
ACEI + CCB	2	5	6	5
ACEI + D	10	12	10	9
αB + ARB	0	1	0	0
αB + βB	1	0	0	0
αB + CCB	1	0	0	0
ARB + βB	1	2	2	0
ARB + CCB	0	1	1	1
ARB + D	2	1	0	0
βB + CCB	3	2	2	3
βB + D	3	2	5	4
CCB + D	4	4	2	2
**3 drugs**	**17**	**14**	**19**	**19**
ACEI + αB + βB	1	2	1	2
ACEI + αB + CCB	1	1	1	1
ACEI + ARB + D	0	0	1	0
ACEI + βB + CCB	2	1	1	0
ACEI + βB + D	3	2	4	4
ACEI + CCB + D	5	5	5	5
αB + ARB + βB	1	0	0	1
αB + βB + CCB	1	1	0	0
αB + CCB + D	0	0	1	1
ARB + βB + CCB	2	1	0	0
ARB + βB + D	0	0	2	3
ARB + CCB + D	0	0	1	2
βB + CCB + D	1	1	2	0
**4 drugs**	**2**	**3**	**3**	**7**
ACEI + αB + βB + CCB	0	1	0	0
ACEI + αB + CCB + D	1	2	1	1
ACEI + βB + CCB + D	1	0	0	2
αB + ARB + βB + D	0	0	0	1
αB + ARB + CCB + D	0	0	1	2
ARB + βB + CCB + D	0	0	1	1
**5 drugs**	**1**	**1**	**0**	**0**
ACEI + αB + βB + CCB + D	1	1	0	0
**All drugs**	**96**	**102**	**90**	**94**

ACE=ACE inhibitor; αB=Alpha blocker; ARB=Angiotensin receptor blocker; βB=Beta blocker; CCB=Calcium channel blocker; D=Diuretic.

Table S2. Antihypertensive medication regimens in the vitamin D-deficient sample; n=150[1]

Antihypertensive regimen	n			
	Vitamin D group		Placebo group	
	Baseline	Follow-up	Baseline	Follow-up
**0 drugs**	**43**	**40**	**39**	**35**
**1 drug**	**15**	**13**	**11**	**19**
ACEI	7	5	5	8
αB	1	1	0	0
ARB	0	1	1	2
βB	2	1	1	3
CCB	2	3	3	5
D	3	2	1	1
**2 drugs**	**7**	**12**	**18**	**16**
ACEI + αB	0	0	0	0
ACEI + ARB	0	0	0	0
ACEI + βB	0	1	2	2
ACEI + CCB	0	3	5	4
ACEI + D	2	3	7	5
αB + ARB	0	0	0	0
αB + βB	0	0	0	0
αB + CCB	1	0	0	0
ARB + βB	0	1	0	0
ARB + CCB	0	1	1	1
ARB + D	1	1	0	0
βB + CCB	1	0	0	1
βB + D	1	1	2	2
CCB + D	1	1	1	1
**3 drugs**	**6**	**5**	**8**	**6**
ACEI + αB + βB	0	0	0	1
ACEI + αB + CCB	0	0	1	1
ACEI + ARB + D	0	0	0	0
ACEI + βB + CCB	1	0	0	0
ACEI + βB + D	1	1	3	1
ACEI + CCB + D	2	3	1	1
αB + ARB + βB	1	0	0	0
αB + βB + CCB	1	1	0	0
αB + CCB + D	0	0	0	0
ARB + βB + CCB	0	0	0	0
ARB + βB + D	0	0	0	1
ARB + CCB + D	0	0	1	1
βB + CCB + D	0	0	2	0
**4 drugs**	**0**	**1**	**3**	**3**
ACEI + αB + βB + CCB	0	1	0	0
ACEI + αB + CCB + D	0	0	1	1
ACEI + βB + CCB + D	0	0	0	1
αB + ARB + βB + D	0	0	0	0
αB + ARB + CCB + D	0	0	1	1
ARB + βB + CCB + D	0	0	1	0
**5 drugs**	**0**	**0**	**0**	**0**
ACEI + αB + βB + CCB + D	0	0	0	0
**All drugs**	**28**	**31**	**40**	**44**

ACE=ACE inhibitor; αB=Alpha blocker; ARB=Angiotensin receptor blocker; βB=Beta blocker; CCB=Calcium channel blocker; D=Diuretic.

Table S3. Eigenvalues of the correlation matrix and loadings in the factor analysis (varimax solution) for change (follow-up minus baseline) in arterial waveform parameters among those with baseline vitamin D deficiency (deseasonalised 25(OH) <50 nmol/L)

		Factor*			
		Factor 1	Factor 2	Factor 3	Factor 4
Eigenvalues of the matrix	Eigenvalue	7.422	2.361	1.651	1.093
	Proportion of total variance	0.530	0.169	0.118	0.078
	Arterial waveform parameter				
Loading (correlation coefficient)	Pulse rate	0.019	**-0.930**	-0.093	0.032
	Brachial SBP	**0.949**	0.118	0.182	0.129
	Brachial DBP	**0.830**	-0.023	-0.064	0.038
	Aortic SBP	**0.930**	0.197	0.245	0.121
	Aortic DBP	**0.911**	-0.196	-0.047	0.049
	Augmentation index	0.058	**0.768**	0.135	0.187
	Pulse wave velocity	**0.926**	0.142	0.219	-0.111
	Peak reservoir pressure	**0.941**	0.265	-0.062	0.123
	Peak excess pressure	0.123	-0.001	**0.976**	-0.049
	Reservoir pressure integral	**0.565**	**0.770**	0.031	0.105
	$\log_e$(excess pressure integral	0.115	0.262	**0.919**	0.119
	Backward pressure amplitude	**0.680**	**0.412**	0.281	**0.443**
	Forward pressure amplitude	**0.812**	0.248	**0.337**	-0.207
	$\log_e$(wave reflection index)	0.043	0.137	0.018	**0.971**

*Factors>0.3 are in bold, which indicates that the variable can be considered a significant constituent of that factor.

Table S4. Changes (follow-up minus baseline) in factor analysis variables (adjusted for age, sex and ethnicity) by treatment group among those with baseline vitamin D deficiency (deseasonalised 25(OH) <50 nmol/L)

Factor*	Mean (standard deviation) change from baseline		Change from baseline, vitamin D minus placebo	
	Vitamin D group (n=71)	Placebo group (n=79)	Mean (95% confidence interval)	P-value†
Factor 1	-0.30 (1.00)	0.22 (1.00)	-0.52 (-0.94, -0.11)	**0.01**
Factor 2	-0.35 (1.00)	0.10 (1.00)	-0.45 (-0.87, -0.04)	**0.03**
Factor 3	0.01 (1.00)	-0.24 (1.00)	0.25 (-0.16, 0.67)	0.23
Factor 4	0.02 (1.00)	0.11 (1.00)	-0.09 (-0.50, 0.33)	0.67

*Described in Table S3.

†Analysis of covariance.

Table S5. Arterial function measures at baseline and follow-up (adjusted for age, sex and ethnicity) by treatment group among those without baseline vitamin D deficiency (deseasonalized 25(OH) ≥50 nmol/L); n=367

Variable	Mean (standard deviation)				Change from baseline, vitamin D minus placebo	
	Vitamin D group (n=185)		Placebo group (n=182)			
	Baseline	Follow-up	Baseline	Follow-up	Mean (95% CI)	P-value
Pulse rate (beats/minute)	63.2 (9.6)	66.0 (9.4)	63.2 (9.5)	66.2 (11.4)	-0.1 (-2.0, 1.8)	0.89
Brachial SBP (mmHg)	138.9 (19.0)	131.0 (16.9)	138.1 (16.0)	131.3 (18.3)	-1.1 (-4.5, 2.4)	0.54
Brachial DBP (mmHg)	78.4 (10.5)	74.1 (10.1)	78.4 (8.9)	74.2 (9.4)	-0.2 (-2.1, 1.7)	0.85
Aortic SBP (mmHg)	141.1 (18.4)	133.3 (16.7)	140.1 (17.4)	132.7 (19.5)	-0.4 (-3.9, 3.1)	0.82
Aortic DBP (mmHg)	72.2 (6.7)	70.0 (6.3)	72.1 (5.4)	70.0 (5.9)	-0.2 (-1.4, 1.1)	0.79
Augmentation index (%)	31.5 (11.5)	29.4 (11.6)	31.6 (12.8)	27.6 (11.8)	2.0 (-0.8, 4.7)	0.16
Pulse wave velocity (m/s)	9.4 (1.6)	9.3 (1.6)	9.4 (1.7)	9.3 (1.8)	-0.0 (-0.2, 0.1)	0.81
Peak reservoir pressure (mmHg)	124.9 (17.7)	118.8 (15.7)	125.0 (15.7)	118.6 (17.6)	0.2 (-3.2, 3.7)	0.90
Peak excess pressure (mmHg)	29.0 (8.8)	25.6 (7.8)	27.6 (8.4)	25.4 (8.5)	-1.2 (-3.2, 0.8)	0.23
Reservoir pressure integral (mmHg.s)	91.7 (18.0)	83.2 (16.8)	92.2 (20.8)	84.1 (20.9)	-0.2 (-4.1, 3.6)	0.91
$\log_e$(excess pressure integral (mmHg.s))	1.59 (0.38)	1.40 (0.41)	1.53 (0.43)	1.35 (0.43)	-0.01 (-0.11, 0.08)	0.77
Backward pressure amplitude (mmHg)	29.0 (7.1)	26.2 (6.4)	29.0 (7.4)	26.0 (7.9)	0.2 (-1.2, 1.6)	0.77
Forward pressure amplitude (mmHg)	40.3 (8.2)	37.6 (8.2)	39.5 (8.4)	37.3 (9.5)	-0.5 (-2.3, 1.3)	0.58
$\log_e$(wave reflection index) ($\times 10^{-2}$)	-119.3 (35.0)	-120.2 (28.2)	-113.0 (33.1)	-122.5 (27.3)	8.7 (-0.0, 17.5)	0.05

SBP=systolic blood pressure; DBP=diastolic blood pressure.

Table S6. Correlations of deseasonalized 25(OH)D concentration (baseline and change*) with changes* in arterial function measures in the vitamin D group

Variable	Correlation coefficient (95% confidence interval)†	
	Baseline 25(OH)‡	Change in 25(OH)§
Pulse rate	-0.03 (-0.28, 0.21)	0.17 (-0.00, 0.33)
Brachial SBP	0.15 (-0.03, 0.34)	-0.23 (-0.43, 0.01)
Brachial DBP	0.18 (-0.02, 0.36)	-0.20 (-0.39, 0.02)
Aortic SBP	**0.21 (0.01, 0.42)**	**-0.25 (-0.42, -0.02)**
Aortic DBP	0.11 (-0.06, 0.28)	-0.13 (-0.33, 0.10)
Augmentation index	**0.18 (0.00, 0.34)**	**-0.23 (-0.39, -0.06)**
Pulse wave velocity	**0.22 (0.02, 0.40)**	**-0.29 (-0.46, -0.08)**
Peak reservoir pressure	**0.24 (0.07, 0.42)**	**-0.31 (-0.48, -0.09)**
Peak excess pressure	-0.12 (-0.32, 0.12)	0.17 (-0.04, 0.35)
Reservoir pressure integral	0.08 (-0.12, 0.29)	**-0.29 (-0.44, -0.11)**
$\log_e$(excess pressure integral	-0.07 (-0.30, 0.17)	0.10 (-0.09, 0.28)
Backward pressure amplitude	0.18 (-0.05, 0.42)	**-0.28 (-0.45, -0.06)**
Forward pressure amplitude	**0.23 (0.02, 0.44)**	**-0.28 (-0.43, -0.09)**
$\log_e$(wave reflection index)	0.06 (-0.18, 0.29)	-0.11 (-0.31, 0.10)

SBP=systolic blood pressure; DBP=diastolic blood pressure.

*Follow-up minus baseline.

†Adjusted for age, sex and ethnicity.

‡Among those with baseline deseasonalized 25(OH)<65 nmol/L.

§Among those with baseline deseasonalized 25(OH)<50 nmol/L (vitamin D deficiency).
